# Mechanisms governing bacterial capsular polysaccharide attachment and chain length

**DOI:** 10.1111/nyas.15364

**Published:** 2025-05-14

**Authors:** Saroj Khadka, Emily L. Kinney, Brooke E. Ryan, Laura A. Mike

**Affiliations:** ^1^ Department of Medicine/Division of Infectious Diseases University of Pittsburgh Pittsburgh Pennsylvania USA; ^2^ Department of Medical Microbiology and Immunology University of Toledo College of Medicine and Life Sciences Toledo Ohio USA

**Keywords:** bacterial glycans, bacterial virulence, capsular polysaccharide, capsule, CPS, EPS, extracellular polysaccharide

## Abstract

Capsular polysaccharides (CPSs) are high‐molecular weight glycopolymers that form a capsule layer on the surface of many bacterial species. This layer serves as a crucial barrier between bacteria and their environment, protecting them from host immune responses and environmental stressors while facilitating adaptation to host niches. The capsule also affects other critical virulence factors of plant and human pathogens such as biofilm production and exchange of antimicrobial‐resistance genes. Bacterial pathogens modulate several CPS properties including abundance, chain length, and cell surface retainment to optimize niche‐specific fitness. CPS composition varies greatly among bacterial species due to differences in sugar units comprising the polymer. Despite the diversity in composition, three conserved CPS biosynthetic systems are common across bacterial species. Although less explored than CPS polymerization and export, the processes of chain length control and attachment are also broadly conserved among bacterial species. Here, we discuss the common strategies that bacteria use to retain CPS to their cell surface and the mechanisms by which bacteria define and control CPS chain length. Additionally, we highlight the outstanding questions related to these processes, identifying areas where future research is needed to gain better insights into these crucial CPS systems.

## INTRODUCTION

Bacterial extracellular polysaccharides (EPSs) are a common structural feature of many bacterial species. Among these, capsular polysaccharides (CPSs or *capsule*) are prominent exopolysaccharides that collectively form a protective layer on the surface of many bacterial pathogens. Many members of both Gram‐positive and Gram‐negative groups are potent producers of CPS. Some notable examples include *Acinetobacter baumannii*, *Burkholderia pseudomallei*, *Haemophilus influenzae*, *Klebsiella pneumoniae*, *Neisseria meningitidis*, *Pseudomonas aeruginosa*, *Staphylococcus aureus*, and *Streptococcus pneumoniae*.^1^, ^2^, ^3^, ^4^, ^5^, ^6^, ^7^, ^8^

Structurally, bacterial capsules consist of long, linear, or branched chains of homo‐ or heteropolysaccharides, which can extend up to 300–400 nm in some species such as *Escherichia* *coli* and *K. pneumoniae*.^9^, ^10^ Furthermore, CPSs are remarkably diverse in composition and antigenicity, with some bacterial genera expressing more than 80 serotypically distinct types of capsule.^3^ Synthesis of these diverse, high‐molecular weight bacterial polysaccharides involves biosynthetic machinery ranging from relatively simple to highly complex systems, which are discussed in detail later in the review.

Capsules and related EPSs serve crucial functions in bacterial pathogens of humans, animals, and plants.^11^, ^12^, ^13^, ^14^, ^15^ For instance, the capsule of *S. pneumoniae* is vital for its survival and escape from vascular endothelial cells, leading to invasive infections.^16^ Similarly, capsule trading among *K. pneumoniae* strains is associated with an increased acquisition of mobile genetic elements, including antibiotic‐resistance and virulence‐associated genes.^17^, ^18^ Noncapsulated forms of pathogens *Pasteurella multocida* in animals and *Erwinia amylovora* in plants show significantly reduced virulence and lack the ability to spread within the host.^12^, ^15^ Additionally, CPS production also influences other bacterial virulence factors, such as biofilm formation in *Vibrio vulnificus* and *Bacteroides thetaiotaomicron*.^19^, ^20^

Capsulated bacteria possess the ability to fine‐tune several aspects of their CPS. Regulation of CPS abundance (i.e., the total amount of capsular material synthesized) through spontaneous mutations in the CPS biosynthesis locus or via transcriptional and translational regulation is common in capsulated bacteria.^21^, ^22^, ^23^, ^24^, ^25^, ^26^ Beyond abundance, bacteria can also manipulate their CPS chain length and its retention on the cell surface, significantly impacting their survival and virulence. For instance, *K. pneumoniae* modulates CPS chain length upon exposure to human urine or nutritionally rich growth medium, consequently altering the mucoviscosity of the bacteria.^27^ Importantly, increased mucoviscosity reduces attachment to phagocytes and association with host epithelial cells.^21^, ^27^, ^28^ Meanwhile, the deliberate release of the anchored CPS from the *S. pneumoniae* cell surface is necessary to breach the host respiratory epithelial cells.^29^, ^30^ By fine‐tuning these more subtle CPS properties, bacteria can effectively promote invasiveness, evade detection, resist phagocytosis, and improve niche‐specific fitness.

Uncovering the mechanisms involved in CPS anchoring and chain length modulation, along with the underlying regulatory processes, is critical for understanding how bacteria fine‐tune their cell surfaces to adapt and persist. While CPS biosynthesis processes, including assembly and export, have been well studied and reviewed, there remains gaps in our understanding about how bacteria control cell‐surface CPS retention and modulate their CPS chain length. Here, we have synthesized our current understanding regarding systems governing bacterial CPS chain length, attachment and release, and present some outstanding questions in the field.

## BACTERIAL CAPSULAR POLYSACCHARIDE BIOSYNTHETIC SYSTEMS

**FIGURE 1:**
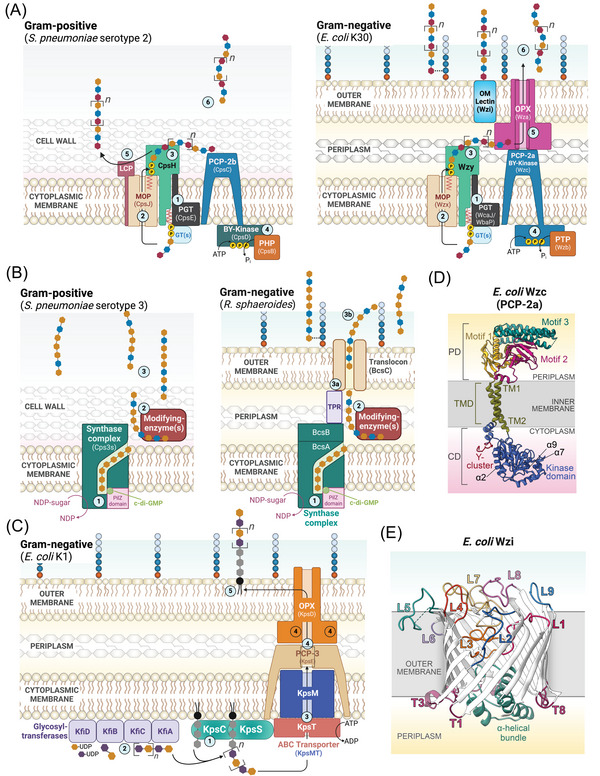
Models of capsular polysaccharide biosynthesis systems in Gram‐positive and Gram‐negative bacteria. (A) Wzy‐dependent systems: Wzy‐dependent capsule synthesis starts with sequential transfer of sugars to a membrane‐linked lipid carrier (Und‐P) by PGT and sugar‐specific GTs to form a sugar repeat unit (①). The lipid carrier is flipped to the periplasmic face by a MOP flippase (②), where the putative Wzy polymerase then progressively adds new sugar repeat units to a growing chain (③). Polymerization rate and final chain length is determined by autophosphorylation and dephosphorylation activity of a PCP‐2a/b and a PTP protein, respectively (④). In Gram‐positive bacteria, the glycan is attached to peptidoglycan by LCP proteins (⑤) or released into the environment (⑥). In contrast, Gram‐negative bacteria require an additional OPX protein for export across the outer membrane (OM) (⑤), and the glycan chains are retained on the surface by multiple strategies including interaction with LPS core and OM lectin (⑥). (B) Synthase‐dependent systems: Synthesis is activated by c‐di‐GMP binding to the PilZ domain of the synthase complex (①). Synthase‐dependent polymers are usually further processed by modifying‐enzymes (②). In Gram‐positive bacteria, the polysaccharide is directly ligated to peptidoglycan or released as a free polymer following the synthesis (③). In Gram‐negative bacteria, the final product is directed to an OM β‐barrel translocon by the TPR domain of the synthase (③a), followed by either release or retention on the cell surface (③b). (C) ABC transporter‐dependent systems: Glycan synthesis begins with the formation of a glycolipid terminus (①). Sugar‐specific glycosyltransferases then transfer sugar repeat units to the lipid acceptor, forming a glycan chain (②). The polysaccharide is translocated across the inner membrane by an ABC transporter KpsMT (③). PCP‐3 protein and OM OPX proteins facilitate translocation across the periplasm and OM (④). The capsular polysaccharide is inserted into the outer membrane via its glycolipid anchor (⑤) or associated with the OM by ionic interactions with LPS or the OPX protein. (D) *E. coli* Wzc monomer: A typical PCP‐2a monomer is composed of periplasmic domain (PD), transmembrane domain (TMD) and cytoplasmic domain (CD). CD α‐helices, α2, α7, and α9 interact with adjacent monomers. The catalytic function of the kinase domain is mediated by the phosphorylation of the C‐terminal tyrosine cluster (Y‐cluster). The structure was obtained from the Protein Data Bank (PDB; 7NHS). (E) *E. coli* Wzi: A 3D structure of *E. coli* Wzi with its extracellular loops (L1–L9), periplasmic turns (T1–T8) and α‐helical bundle color coded. The structure was obtained from Protein Data Bank (PDB; 2YNK). _BY‐Kinase, bacterial tyrosine kinase; GT, glycosyltransferase; LCP, LytR‐CpsA‐Psr; LPS, lipopolysaccharide; MOP, multidrug/oligosaccharidyl‐lipid/polysaccharide; OM, outer membrane; OPX, outer membrane polysaccharide export; PCP, polysaccharide co‐polymerase; PGT, phospho‐glycosyltransferase; PTP, protein tyrosine phosphatase; UndP, undecaprenyl‐phosphate; TM1, transmembrane 1; TM2, transmembrane 2; TPR, tetratricopeptide repeat.

CPSs are diverse macromolecular structures with many bacterial species, such as *S. pneumoniae*, *K. pneumoniae*, and *E. coli*, each known to synthesize over 80 serotypically and structurally distinct capsule types.^3^, ^31^, ^32^ Despite considerable diversity in CPS composition, the mechanisms and proteins driving CPS biosynthesis are relatively conserved across CPS‐producing bacterial species. At present, there are four main EPS biosynthetic pathways in bacteria; these include (i) Wzy‐dependent, (ii) ATP‐binding cassette (ABC) transporter‐dependent, (iii) synthase‐dependent, and (iv) extracellular transglycosylase‐dependent mechanisms.^31^, ^33^, ^34^ While the latter is involved in extracellular biosynthesis of glycans that function in surface adhesion (e.g., *Streptococcus mutans* dental plaque biofilms), the three former systems are involved in the biosynthesis of the protective CPS.^34^ The Wzy‐ and synthase‐dependent mechanisms are used by both Gram‐positive (e.g., *S. pneumoniae*) and Gram‐negative (e.g., *E. coli*) bacteria, while the ABC transporter‐dependent method has only been described in Gram‐negative bacteria (e.g., *E. coli* K1 and K5, *Salmonella enterica* serovar Typhi).^3^ A brief overview of these three CPS biosynthetic systems follows, but see Whitfield et al. 2006 and 2020 for more in‐depth reviews.^31^, ^34^ A graphical illustration of the CPS biosynthetic systems discussed below is presented in Figure 1.

### Wzy‐dependent systems

The Wzy‐dependent system is the predominant CPS biosynthesis system found in Gram‐positive and Gram‐negative bacteria, representing over 70% of total capsulated prokaryotes.^35^ Almost all the *S. pneumoniae* CPSs (except serotype 3 and 37) and *E. coli* Group 1 and 4 CPSs are synthesized by this mechanism. The Wzy‐dependent pathway shares significant similarity with Gram‐negative lipopolysaccharide (LPS) O‐antigen biosynthesis.

Most of our current understanding about Wzy‐dependent mechanism comes from the study of the prototypical *E. coli* Group 1 CPS biosynthesis pathway. The Wzy‐dependent CPS synthesis is initiated by a polyprenol phosphate phospho‐glycosyltransferase (PGT; e.g., WbaP or WcaJ in *E. coli* and CpsE in *S. pneumoniae*) that transfers the first sugar of the sugar repeat unit to a membrane‐bound undecaprenyl phosphate (Und‐P) lipid carrier.^34^, ^36^, ^37^ Additional serotype‐defining glycosyltransferases (GTs) add other nucleotide sugars to complete the formation of a complete sugar repeat unit.^37^ The sugar‐Und‐PP repeat unit is flipped across the inner membrane to the periplasmic surface by a multidrug/oligosaccharidyl‐lipid/polysaccharide (MOP) family transporter (e.g., Wzx in *E. coli*, and CpsJ in *S. pneumoniae*). Polymerization is achieved by the coordinated function of the polymerase (e.g., Wzy in *E. coli*, and CpsH in *S. pneumoniae*), chain length‐regulating PCP‐2a with intrinsic tyrosine‐kinase activity or PCP‐2b associated with a separate tyrosine kinase (e.g., Wzc in *E. coli*, and CpsCD in *S. pneumoniae*) and its cognate protein tyrosine phosphatase (PTP; Wzb in *E. coli*, and CpsB in *S. pneumoniae*). Upon completing polymerization, Gram‐negative CPS chains are exported through an outer‐membrane polysaccharide export (OPX) protein (Wza in *E. coli*) which also requires an interaction with the PCP‐2a/b.^38^, ^39^ Gram‐positive CPS is either retained on the cell surface by LytR‐CpsA‐Psr (LCP) family proteins ligating CPS to peptidoglycan or released as EPS by an unknown mechanism.^40^, ^41^, ^42^ In contrast, the exact mechanism of CPS retention is not known in Gram‐negative bacteria with some studies suggesting involvement of the lectin protein Wzi and ionic interactions between CPS and LPS.^9^, ^43^, ^44^ Current knowledge regarding attachment of Wzy‐dependent CPS is detailed below in “Mechanism and regulation of capsular polysaccharide chain length” section.

### Synthase‐dependent systems

The production of *S. pneumoniae* serotype 3 and 37 capsules, *Streptococcus pyogenes* hyaluronan capsule and the capsule‐like alginate polysaccharide of *Pseudomonas aeruginosa* are a few examples of CPSs synthesized by the synthase‐dependent mechanism.^34^, ^45^, ^46^ The CPSs or EPSs synthesized by the synthase‐dependent mechanism are relatively simple in structure, composed of just one or two sugar units. This mechanism poses two striking differences from other CPS biosynthesis mechanisms, (a) the synthase method uses fewer proteins (typically one or two) to carry out all key steps of CPS biosynthesis including initiation, polymerization and transport of the CPS chain, and (b) the synthase enzyme typically has a processive polymerase activity, which instead of a distinct sugar repeat unit, directly adds individual sugars to a growing polysaccharide chain.

Both Gram‐negative and Gram‐positive bacteria employ a synthase complex that is a glycosyltransferase‐2 (GT2) family β‐glycosyltransferase protein with processive enzymatic activity. In Gram‐negative bacteria, a β‐barrel transmembrane translocon found in the bacterial outer membrane interfaces with the synthase complex via a periplasmic tetratricopeptide repeat (TPR)‐containing scaffold domain.^47^ Despite some variations in the synthase complexes and its regulation, the core biosynthetic steps remain the same between different synthase‐dependent CPS biosynthesis mechanisms. The Gram‐negative BcsAB cellulose synthase is a well‐studied EPS synthase prototype. The membrane‐spanning cellulose synthase complex in *Rhodobacter sphaeroides* contains an inner membrane BcsA catalytic subunit and periplasmic BcsB subunit that is likely involved in directing the nascent polysaccharide to the outer membrane.^48^ BcsA is a GT2 enzyme and its transmembrane helices form the cellulose translocon in the inner membrane. Binding of cyclic di‐GMP (c‐di‐GMP) to the PilZ domain of BcsA activates its glycosyltransferase activity. Additional EPS‐modifying enzymes have been reported to further modify the synthase‐dependent CPS following polymerization, varying surface electrostatics and polymer solubility.^49^, ^50^ In Gram‐negative bacteria, the BcsC β‐barrel translocon facilitates the final polymer export across the outer membrane.^51^ BcsC is predicted to interact with BcsB via a BcsC periplasmic TPR domain during synthesis and export.^51^

*S. pneumoniae* employs synthase‐dependent machinery to produce serotypes 3 and 37 CPS.^45^ The serotype 3 CPS is composed of alternating glucose and glucuronic acid units, and the serotype 37 CPS is a linear chain of glucose with side chains of additional glucose units.^3^, ^52^, ^53^ The serotype 3 CPS synthesis is initiated by a type 3 synthase (Cps3S/Cap3b) when it transfers glucose from an undecaprenyl phosphate‐glucose (UDP‐Glc) unit to a phosphatidylglycerol (PG) acceptor present in the bacterial membrane.^54^ The use of a lipid acceptor differentiates *S. pneumoniae* synthase‐dependent CPS biosynthesis from the majority of other synthase‐dependent systems. Once eight sugar residues are added to form an oligosaccharide, the chain is either released from the synthase or gets further extended to form a polysaccharide chain.^55^ The polysaccharide chain is extended by alternating addition of glucuronic acid (GlcA) and glucose at the nonreducing end of the growing chains. Dissociation and determination of CPS polymer chain length is dictated by intracellular concentrations of UDP‐sugars (see “Intracellular pool of nucleotide‐sugar precursors modulates CPS chain length in synthase‐dependent systems” section).^56^ The synthase‐dependent CPSs are either noncovalently retained on the cell surface or released to the environment.

### ABC transporter‐dependent systems

Primarily described in Gram‐negative bacteria, the ABC transporter‐dependent mechanism is the second most common CPS assembly and export system.^35^ *E. coli* K1 and K5 CPSs and *Salmonella* Typhi Vi‐antigen CPS are three prominent examples of CPSs synthesized by ABC transporter‐dependent pathways. The use of an ABC transporter to translocate the glycolipid CPS across the inner membrane is characteristic of this mechanism.

*E. coli* Group 2 and 3 CPS biosynthesis machinery are prototypical ABC transporter‐dependent systems. The *E. coli* proteins involved in this mechanism are encoded by the *kps* locus, where the KpsM (nucleotide‐binding domain) and KpsT (transmembrane domain [TMD]) complex forms the ABC transporter.^34^, ^57^ CPS synthesis is initiated by sugar‐specific GTs and may require special transition transferases to add a CPS primer.^58^ The GTs transfer individual sugar units to the nonreducing end of the polymer of β‐linked 3‐deoxy‐d‐*manno*‐oct‐2‐ulosonic acid (poly(Kdo)) attached to a PG acceptor.^59^ The use of poly(Kdo) as a linker between the lipid acceptor and polysaccharide is characteristic of the ABC transporter‐dependent CPSs with a notable exception in *Salmonella* Typhi Vi‐CPS biosynthesis, which lacks the ability to synthesize the poly(Kdo) linker.^60^, ^61^ Instead, the linker of *S*. Typhi Vi‐CPS is composed of an *N*‐acetylhexosamine modified with two β‐hydroxy chains that closely resembles the hydrophobic portion of LPS lipid A.^61^ Once glycolipid‐CPS synthesis is completed, the ABC transporter (KpsMT) recognizes the conserved glycolipid terminus of the CPS, reorients the lipid anchor to the periplasmic leaflet of the inner membrane then expels the CPS component through the inner membrane to the outer membrane translocon formed by PCP‐3 and OPX proteins. The movement of CPS is likely driven by conformational rearrangements and rigid‐body motion of the ABC transporter nucleotide‐binding domain in KpsM.^34^, ^62^ It is currently unclear whether the lipid moiety is extracted from the inner membrane and integrated into the outer membrane or if it remains in the inner membrane.^62^ Finally, the polysaccharide synthesized by the ABC‐dependent transporter is translocated across the outer membrane through a channel formed by the PCP‐3 and OPX proteins. The CPS chain length is presumed to be determined by the combined function of synthesis and export and warrants further investigation.^34^, ^63^ Furthermore, ABC transporter‐dependent CPS may be retained on the cell surface by anchoring the lipid‐attached CPS into the bacterial membrane, ionic interactions between CPS and LPS, or close association with the OPX protein (see “Mechanism and regulation of capsular polysaccharide chain length” section).^10^, ^59^, ^61^, ^64^, ^65^, ^66^

## MECHANISM AND REGULATION OF CAPSULAR POLYSACCHARIDE ATTACHMENT

Capsules affect bacterial fitness by providing protection against biotic and abiotic stress and by modulating adhesion to different surfaces, such as host cells. While CPSs are tightly associated with the bacterial cell surface following synthesis, the polysaccharides can even affect bacterial survival and adherence when dissociated from the bacterial surface. For example, unattached capsules can still inhibit *K. pneumoniae* opsonophagocytosis and protect *Bifidobacterium breve* against abiotic stresses, such as low pH and bile acids.^67^, ^68^ Furthermore, adding purified CPS to an inoculum of *B. pseudomallei* increases the survival of the bacteria in the blood and increases the number of bacterial cells recovered by allowing the bacteria to evade complement.^69^

Decreased CPS attachment or active capsule shedding alters bacterial interactions with host cells, consequently impacting pathogenesis. In *S. pneumoniae*, active capsule shedding mediated by the autolysin LytA is induced by host antimicrobial peptides during the early stages of acute lung infection.^29^ This shedding enhances interactions with host epithelial cells and promotes bacterial invasion.^29^ Meanwhile, an unattached but closely associated capsule can behave as an exopolysaccharide layer, preventing adherence of *K. pneumoniae* and *S. pneumoniae* to phagocytes and epithelial cells.^27^, ^70^ As a result, this unattached, but cell‐associated capsule reduces the ability of *S. pneumoniae* to translocate from the primary site of infection in a murine pneumococcal infection model.^70^ *Neisseria meningitidis* sheds its capsule to better invade host epithelial cells.^71^ CPS attachment and release appear to be a regulated process in some bacteria. Shedding capsule during an infection can reduce the amount of encapsulated bacteria present and effectively remove host defense components from the cell surface.^29^ For example, *K. pneumoniae*, *S. pneumoniae*, and *P. aeruginosa* release capsules after encountering antimicrobial peptides, and this released capsule neutralizes the bactericidal activity of the antimicrobial peptides.^72^ Likewise, unattached *Bacillus anthracis* capsules can contribute to resistance to cationic antimicrobial peptides (CAMPs).^73^ The shed capsule can also interfere with the immune system. In *B. anthracis*, shed capsule can inhibit the function of dendritic cells and increase pathogenicity.^74^, ^75^

**FIGURE 2:**
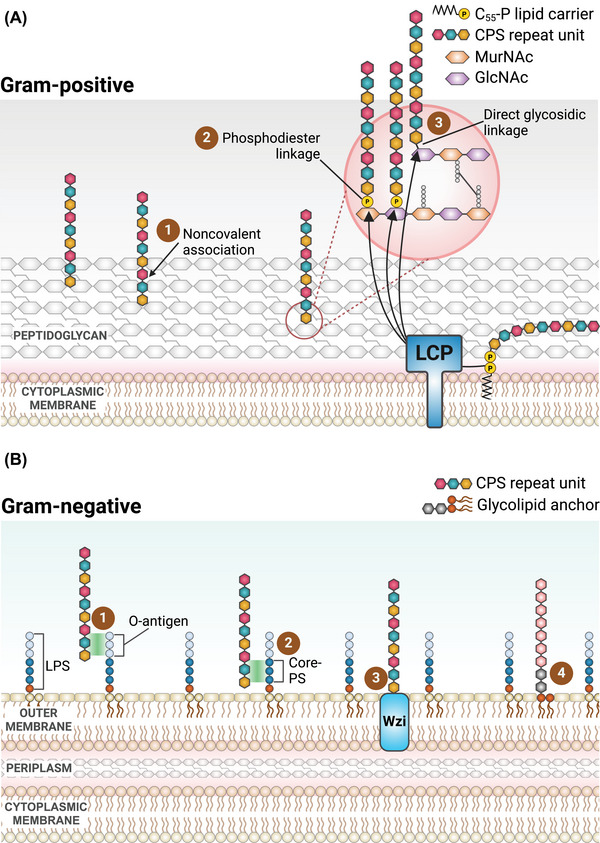
Mechanisms of capsular polysaccharide (CPS) attachment in Gram‐positive and Gram‐negative bacteria. (A) Gram‐positive CPS can be (①) noncovalently or (②, ③) covalently retained by the thick peptidoglycan layer. CPSs are covalently ligated to the peptidoglycan by the LytR‐Cps2A‐Psr (LCP) class of enzymes by forming either a (②) phosphodiester linkage or (③) direct glycosidic linkage with *N*‐acetylglucosamine (GlcNAc) or *N*‐acetylmuramicacid (MurNAc) of peptidoglycan. (B) In Gram‐negative bacteria, CPS can be held on the outer membrane surface by ionic interactions with the (①) O‐antigen or (②) core‐polysaccharide (PS) of LPS, and by interactions with the cell surface lectin (③) Wzi or using a (④) lipid anchor.

CPS association and release from the cell surface are driven by distinct mechanisms in Gram‐positive and Gram‐negative bacteria. In Gram‐positive bacteria, CPS is usually linked to peptidoglycan by the action of LCP enzymes. Since peptidoglycan is not exposed in Gram‐negative bacteria, these species strongly retain their capsules on the cell surface by anchoring CPS to the outer membrane lectin Wzi, lipids, and LPS structural domains. In this section, we will discuss several known and potential mechanisms of CPS attachment described in Gram‐positive and Gram‐negative bacteria, as well as mechanisms regulating CPS attachment and release. An overview of the different CPS attachment mechanisms and regulatory factors are presented in Figure 2.

### Capsular polysaccharide attachment in Gram‐positive bacteria is commonly facilitated by the LytR‐CpsA‐Psr (LCP) class of enzymes

The LytR‐CpsA‐Psr (LCP) class of phosphotransferases serves a general role in maintaining Gram‐positive bacterial cell wall integrity.^76^ LCPs catalyze the formation of phosphodiester bonds between cell wall glycopolymers and peptidoglycan constituents *N*‐acetylmuramicacid (MurNAc) or *N*‐acetylglucosamine (GlcNAc). LCP proteins have been implicated in attaching different anionic cell wall glycopolymers, such as CPS and wall teichoic acids, to peptidoglycan in Gram‐positive bacterial species, including *Staphylococcus* and *Streptococcus* species.^41^, ^77^, ^78^ In *S. pneumoniae*, the LCP proteins Cps2A, LytR, and Psr ligate CPS chains to peptidoglycan.^40^ Similar function is ascribed to CpsA in *S. agalactiae* and LcpC in *S. aureus*.^41^, ^79^ LCP genes are usually located near the genes required for glycopolymer synthesis (e.g., *cps2A* is the first gene in *S. pneumoniae* serotype 2 CPS biosynthesis locus), coupling polymer synthesis and export to covalent cell‐surface attachment.^76^

The well‐characterized *S. pneumoniae* Cps2A is a prototypical example of LCP protein structure and function. An LCP protein typically contains three major domains: (i) a TMD, (ii) a nonconserved accessory domain, and (iii) the core catalytic LCP domain. The core LCP domain has an α‐β‐α architecture where six α‐helices surround the catalytic core formed by five β‐sheets.^76^ Cps2A has polyprenol pyrophosphatase activity. Its core LCP domain binds polyprenoid pyrophosphate lipid substrates in its hydrophobic pocket, such as the Und‐PP lipid component of a lipid‐bound CPS.^40^, ^76^ However, it appears to have no specificity toward the polysaccharide component of the CPS, as the protein has space enough to accommodate only the lipid carrier and a sugar moiety directly attached to the lipid.^76^ This nonspecificity toward carbohydrates while maintaining strong affinity with the lipid component likely allows the LCP proteins to anchor varying serotypes of CPSs to peptidoglycan. For example, several *S. pneumoniae* serotypes are shown to be covalently linked to peptidoglycan.^80^ These *S. pneumoniae* serotypes encode *cps2A* homologs in the CPS biosynthetic locus.^3^

Although *S. aureus* lacks an obvious *cpsA* or *cps2A* homolog in the CPS biosynthesis locus, it encodes for three other LCP proteins with semiredundant functions: LcpA, LcpB, and LcpC. Among the three, LcpC encodes the key enzyme required to anchor *S. aureus* CP5 CPS to cell wall peptidoglycan.^4^, ^41^ Meanwhile, LcpA and LcpB play a major role in attachment of wall teichoic acid to peptidoglycan. Nonetheless, a complete release of the capsule from the surface requires deletion of all three LCP proteins, indicating some cross‐functionality.^41^ *S. aureus* LcpC cleaves CPS from its lipid carrier and ligates it to the glycan strand of peptidoglycan likely via a phosphodiester linkage.^4^, ^41^ In vitro studies suggest that the ultimate peptidoglycan precursor lipid II_PG_ serves as the CPS acceptor.^4^

The role of LCP proteins in CPS attachment has only been well‐established for *Staphylococcus* and *Streptococcus*. However, LCP proteins are widespread in Gram‐positive bacterial species, and it remains to be seen whether these LCP proteins functionally contribute to anchoring CPS in other Gram‐positive bacteria.

### Wzi is a unique lectin that partially retains the Gram‐negative Group 1 capsular polysaccharide on the outer membrane

Wzi is a Gram‐negative outer membrane lectin associated with the Wzy‐dependent pathway of some Group 1 CPS‐producing bacterial species. The occurrence and functional importance of Wzi in CPS attachment has been studied for *E. coli*, *K. pneumoniae*, and *A. baumannii*.^9^, ^43^, ^44^, ^81^ *wzi* is typically encoded within the 5′ upstream region of CPS biosynthesis locus, except in *A. baumannii*, where it is located outside of the CPS biosynthesis locus.^81^ Wzi homologs are primarily found in the *Proteobacteria* phylum, but are sporadically present in other phyla as well.^81^ Wzi exhibits some sequence diversity among bacterial isolates, which in *K. pneumoniae* strongly correlates with its capsule type and facilitates genome‐based capsule‐typing of *K. pneumoniae*.^82^ On the contrary, sequence diversity analysis shows no such association between the *wzi* locus and capsule type in *A. baumannii*.^81^

Deleting *wzi* reduces cell‐surface CPS retention by approximately 40%–50% in *K. pneumoniae*, while a similar mutant in *A. baumannii* has 75%–80% less CPS retention.^9^, ^44^, ^81^ The remaining cell‐associated CPS after Wzi deletion is unable to form a consistent capsular layer and appears as sparse patches of polysaccharides on the surface of *E. coli* and *A. baumannii*.^44^, ^81^ Wzi deletion strains exhibit no defect in CPS synthesis, indicating that the effect of Wzi on CPS attachment does not provide feedback to CPS biosynthesis.

Most of our current understanding of Wzi comes from the analysis of the prototypical Wzi of *E. coli* K30. Structurally, the Wzi protein is a monomeric, 18‐stranded antiparallel β‐barrel protein featuring an inner channel with a 36 Å diameter.^43^, ^44^, ^83^ The β‐strands on the extracellular side are connected by 9 loops (L1–L9) which extend outward or fold inward to the channel (Figure 1E).^43^ On the periplasmic side, the β‐strands are connected by eight periplasmic turns (T1–T8; Figure 1E).^43^ Furthermore, the C‐ and N‐termini, including an α‐helical bundle preceding the N‐terminus are exposed to the periplasm. In molecular dynamics (MD) simulations, the L5 loop extends outward from the Wzi barrel and inserts into the membrane lipid bilayer via hydrophobic, hydrogen bonding and electrostatic interactions.^84^ Similarly, the exposed hydrophobic residues of L6 are also suggested to be inserted into the membrane.^43^ The conserved cationic residues on the loops L3, L6, and L7 appear to provide sites for electrostatic interactions with the anionic CPS as individual deletion of L3, L6, or L7 results in CPS release from the cell surface.^43^, ^81^ Similarly, deletion of the periplasmic α‐helical bundle also causes increased CPS release in *E. coli* suggesting a role of these periplasmic α‐helices in CPS attachment.^43^

### Interactions between CPS and LPS structural domains facilitate capsule retention to the Gram‐negative cell surface

LPS is another important Gram‐negative cell–surface‐associated exopolysaccharide. The prototypical LPS structure is composed of an O‐antigen polysaccharide, a core oligosaccharide, and a lipid A anchor. Not all encapsulated Gram‐negative bacterial species encode Wzi. Moreover, in Wzi‐encoding strains, Wzi alone does not ensure complete CPS retention as deleting Wzi does not release all cell‐associated CPS into the environment.^9^, ^44^, ^81^ Thus, an auxiliary CPS attachment mechanism, where the CPS is noncovalently linked to LPS, has been proposed based on studies in *E. coli* and *K. pneumoniae*.^9^, ^85^, ^86^

Both the LPS O‐antigen and core oligosaccharide have been linked to Gram‐negative capsule retention, although conclusions have differed.^9^, ^87^ O‐antigen is connected to lipid A‐core oligosaccharide by the O‐antigen ligase, WaaL. Deleting WaaL significantly reduced cell–surface‐associated *K. pneumoniae* (O1:K2) CPS in a study by Singh et al. based on cell‐associated uronic acid content; however, Fresno et al. observed no defect in K2 CPS retention in *K. pneumoniae waaL* (O1:K2) based on phage sensitivity assays and ELISA using K‐specific antibodies.^9^, ^85^ Similarly, an O‐antigen ligase mutant in *E. coli* did not reduce surface‐associated CPS based on sedimentation resistance.^86^ It is possible that the different techniques used to quantify cell‐associated CPS could have resulted in different conclusions from these independent groups.^9^, ^85^ Thus, further studies are required to resolve the exact role of LPS O‐antigen in CPS retention.

In contrast, the core oligosaccharide has been repeatedly reported to partially contribute to capsule retention in *K. pneumoniae*. The core oligosaccharide‐associated galacturonic acid (GalA) moieties are required for complete retention of *K. pneumoniae* K2 capsule.^85^ In *K. pneumoniae*, GalA transferases WabO and WabG catalyze the transfer of two GalA sugars to the LPS core. Deletion of *wabO* or *wabG* reduces the cell‐associated capsule by up to 90%.^85^, ^87^ It is proposed that the CPS is retained on the bacterial surface via a cationic bridge (Mg^2+^ and/or Ca^2+^) between the anionic CPS and negatively charged core oligosaccharide.^85^, ^88^, ^89^ This is based on observations that either increasing the ionic strength of or depleting metal ions from culture medium dissociates CPS from the cell surface.^85^ Moreover, divalent cations within the LPS inner core region are critical for mitigating repulsive forces between negatively charged LPS polymers and maintaining outer membrane integrity and stability. Some LPS variants such as those found in *E. coli*, *S. enterica*, and *P. aeruginosa* lack acidic sugars. In such cases, the negatively charged phosphates in the LPS inner core may serve as a site of interaction for anionic CPS likely bridged by a cation.^90^, ^91^ However, whether the phosphates are accessible for interaction with CPS remains unknown. Metal‐dependent ionic bonds between the relatively conserved core oligosaccharide structure and typically anionic CPS (despite greater diversity in composition) could explain how Gram‐negative species anchor a wide variety of capsular serotypes to the bacterial cell surface.

### Conserved glycolipid terminus attaches ABC transporter‐dependent capsular polysaccharide to the Gram‐negative surface

ABC transporter‐dependent CPS systems initiate CPS polymerization on a conserved glycolipid terminus composed of β‐Kdo and PG. Two enzymes, KpsS and KpsC (CtrE and CtrF in *N. meningitidis*), conserved across Gram‐negative ABC transporter‐dependent systems, synthesize a poly‐Kdo linker that bridges PG to the CPS. Specifically, KpsS transfers a β‐Kdo residue to a PG acceptor, and KpsC subsequently extends the β‐Kdo linker. Treating highly purified CPSs from *E. coli* K1 and K5 and *N. meningitidis* group B with capsule‐specific bacteriophages provided evidence of this attachment.^59^, ^92^ The resulting depolymerized products included an intact lipid terminus with initial CPS residues linked by a poly(Kdo) linker.^59^ Although *S*. Typhi Vi‐antigen CPS is synthesized using a similar ABC transporter‐dependent system, it lacks KpsS and KpsC homolog. Instead, the Vi‐antigen CPS is attached to a modified glycolipid carrier, diacyl‐HexNAc by the enzyme VexE.^61^ The *S*. Typhi VexE deletion mutant releases a significantly large amount of Vi‐antigen to the growth medium in vitro compared to the wild‐type strain, supporting the role of glycolipid anchor in CPS attachment.^61^ Furthermore, observations from super‐resolution fluorescence microscopy (STORM) suggest that the ABC transporter‐dependent glycolipid‐CPS forms aggregated region referred to as “capsular rafts” which are localized near the CPS export channel.^10^ Furthermore, the rafts are suggested to spread out gradually as more glycolipid‐CPS products are exported from the CPS export channel.^10^ In addition to CPS attachment, the glycolipid terminus acts as a signal for binding and translocation through the ABC transporter.^93^ Despite the mounting evidence suggesting the role of the glycolipid terminus in CPS attachment, the molecular mechanism by which the terminus is transported from the inner membrane to the outer membrane is not fully understood.

### Regulation of capsular polysaccharide attachment to the cell surface is undefined

The regulation of CPS attachment is not as widely reported. In *S. pneumoniae*, exposure to the CAMP LL‐37 induces CPS release from the cell surface.^29^ This process is mediated by the streptococcal autolysin LytA.^29^ LytA cleave the bond linking the peptide stem to N‐acetylmuramic acid in peptidoglycan. Interestingly, LytA activity induced by CAMP is not fatal to bacterial cells, unlike when it is activated by antibiotics.^29^ Further, the gradual increase in CPS detected in the culture supernatant over time suggests that CPS attachment could be an actively regulated process in *S. pneumoniae*.^29^ Similarly, treatment of *K. pneumoniae* and *P. aeruginosa* with the CAMP, human neutrophil α‐defensin 1 (HNP‐1) increases capsule release from the cell surface.^72^

Notably, even in bacterial strains with covalently anchored CPS, some proportion of capsule is steadily released into the growth medium in vitro. However, the specific environmental signals required to induce CPS release and the bacterial factors facilitating this process remain poorly understood. Future research is needed to determine whether capsule release is a dynamic and regulated process during active infection.

## MECHANISM AND REGULATION OF CAPSULAR POLYSACCHARIDE CHAIN LENGTH

Some pathogenic bacteria modulate CPS chain length to effectively optimize fitness and enhance virulence. Variations in CPS chain length can alter bacterial surface‐associated features such as capsule thickness and mucoviscosity, influencing how bacteria interact with their environment. While capsule thickness has been used as a proxy for CPS chain length in the following discussion, it is important to note that capsule thickness may be influenced by multiple factors, including CPS chain length, the organization of CPS chains into layers and the density of CPS chains packed within a unit surface area. Therefore, the relationship between capsule thickness and CPS chain length may not always be direct.

An overall increased CPS abundance is often associated with increased virulence, persistence, and survival in many capsulated bacteria. However, a thicker capsule can be counterproductive to bacteria by masking critical surface‐associated virulence factors required at different stages of bacterial pathogenesis.^94^, ^95^, ^96^ For instance, a thicker Group 4 capsule in *Shigella sonnei* and enterohemorrhagic *E. coli* (EHEC) blocks the type III secretion system (T3SS) components and adhesins from interacting with the target host cells.^97^, ^98^ Since the T3SS is a crucial factor in the invasion of host cells, Group 4 capsule thickness in *S. sonnei* and EHEC is thought to be regulated at different stages of pathogenesis.^97^, ^98^ In enteropathogenic *E. coli* (EPEC) and EHEC, capsule thickness is modulated to maintain a time gap between the translocation of effector molecules into the host cell and exposure of adhesins such as intimin.^98^ Similarly, Group 4 capsule thickness is negatively associated with invasiveness and dissemination potential of *S. sonnei*, but positively correlated with resistance to complement factors.^97^ This highlights the importance of modulating capsule thickness by altering CPS production and/or CPS chain length during various stages of infection. It collectively suggests that dynamic modulation of capsule thickness could provide host environment‐dependent fitness to capsulated bacteria. Similarly, *S. pneumoniae* fine‐tunes CPS chain length and maintains its abundance accordingly depending on the environment.^99^ In low O_2_ environments, *S. pneumoniae* produces shorter but abundant CPS chains that shield vulnerable bacterial regions such as the division septum.

Capsulated bacteria can also modulate CPS chain length to manage the limited supply of critical precursors shared by CPS and other cell surface components. As sugars and Und‐P precursors become scarce, *S. pneumoniae* ensures supply of these precursors to both CPS and cell envelope synthesis by producing longer CPS chains that require less Und‐P without compromising the protective function of capsule.^99^

*K. pneumoniae* serves as another attractive model as the regulation of CPS chain length significantly impacts its virulence and pathogenesis. *K. pneumoniae* with longer, more uniform CPS chains appear hypermucoviscous, which significantly enhances the ability of bacteria to cause invasive diseases.^27^, ^100^, ^101^ Moreover, hypermucoviscous *K. pneumoniae* are associated with decreased phagocytosis by neutrophils and macrophages and have increased resistance to being trapped by neutrophil extracellular traps.^102^ Similarly, hypermucoviscous (indicative of longer CPS chains) *A. baumannii* shows reduced adherence to epithelial cells and reduced internalization by macrophages.^103^ Alternatively, reduced mucoviscosity (indicative of shorter, diverse CPS chains) in *Burkholderia multivorans* leads to decreased adherence to epithelial cells and significantly reduced virulence in a *Galleria mellonella* infection model.^104^

**FIGURE 3:**
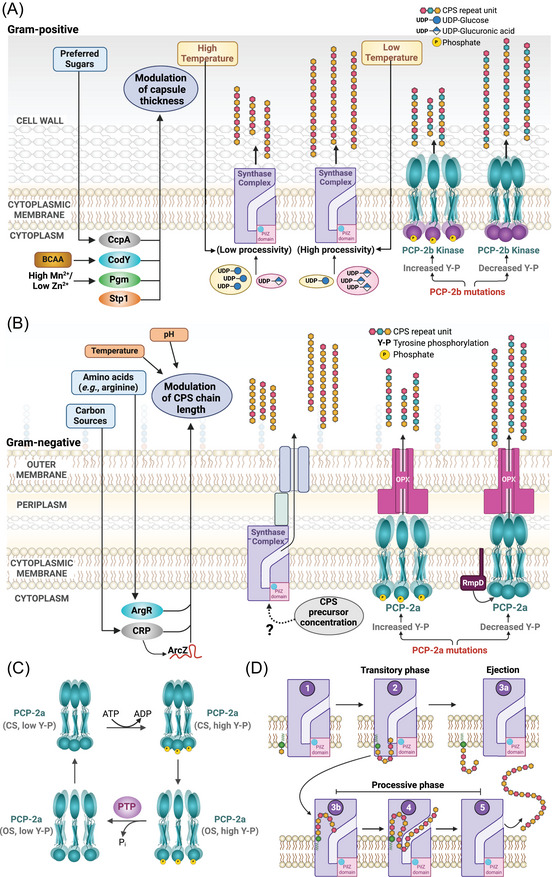
Mechanisms regulating capsular polysaccharide (CPS) chain length in Gram‐positive and Gram‐negative bacteria. (A) In Gram‐positive bacteria, capsule thickness, that is, increased CPS chain length and abundance is regulated by carbon sources. Capsule thickness is influenced by the availability of carbon sources and other environmental signals through global regulators CcpA and CodY, and metabolic factors such as Pgm. Synthase‐dependent CPS is controlled by intracellular CPS precursor concentration. The synthase complex attains a high processivity state at a low temperature and with an increased UDP‐galactose pool. Meanwhile, in Wzy‐dependent system, the phosphorylation status of the PCP‐2b kinase domain regulates CPS chain length. The auto‐phosphorylation dynamics can be modulated by spontaneous acquisition of PCP‐2b genetic mutations. (B) Environmental factors such as carbon sources, temperature, and pH are known to alter Gram‐negative CPS chain length. Specific mechanisms behind this environmental regulation are unclear. However, the global carbon catabolite regulator, cAMP receptor protein (CRP) and the small RNA, ArcZ, as well as the arginine‐responsive transcriptional regulator, ArgR relay information about carbon and nitrogen‐source availability to modulate CPS chain length. Substrate‐dependent CPS chain length regulation in Gram‐negative synthase‐dependent systems has yet to be reported. CPS chain length regulation in Gram‐negative Wzy‐dependent systems is primarily mediated by the autophosphorylation cycle of PCP‐2a, similar to Gram‐positive PCP‐2b. In *K. pneumoniae*, a unique protein, RmpD interacts with PCP‐2a to further fine‐tune CPS chain length by an unknown mechanism. The high and low phosphorylation states can be shifted by spontaneous PCP‐2a mutations. (C) PCP‐2a/b cycles between the closed state (CS) and the open state (OS) depending on the phosphorylation of C‐terminal tyrosine residues. Increased autophosphorylation promotes dissociation of PCP‐2a/b into individual monomers which likely requires crossing a critical phosphorylation threshold. A cognate PTP/PHP dephosphorylates tyrosine residues and promotes PCP2‐a/b complex octamerization. The duration of the CS and the OS and the threshold of phosphorylation required to dissociate the complex regulates the CPS chain length. (D) CPS polymerization by a synthase complex can attain a highly processive state when the polysaccharide is strongly held by the complex, thus synthesizing a long and high‐molecular weight CPS product (steps 3b, 4, 5). In contrast, failure to generate a lipid‐oligosaccharide primer of a critical length (∼8 sugar units) causes the primer to dissociate from the complex (step 3a). Similarly, a high intracellular concentrations of UDP‐glucose ejects the polysaccharide from the binding site of synthase complex and generates shorter CPS chains.

The regulation of capsule thickness influenced by changes in CPS chain length and abundance contributes to *Streptococcus suis* and *S. pneumoniae* virulence.^105^ *S. pneumoniae* capsule shows reduced thickness following the binding to host epithelial cells.^30^ Similarly, capsule thickness as a phenotype has consequences in colonization of the host, IL‐17–dependent clearance of the bacteria, transmission between hosts, NET induction, and levels of bacteremia.^106^, ^107^, ^108^ *S. pneumoniae* with thicker capsules are more effective at repelling mucus and show less binding to upper respiratory tract mucin than their counterparts with thinner capsules.^109^ However, the relationship between pathogenesis and capsule thickness are not always in agreement across different reports. A study on global regulators of *S. suis* identified mutants producing thinner or thicker capsules compared to the wild‐type parental strain.^110^ Interestingly, both thin‐ or thick‐capsule mutants exhibited reduced adherence to and invasion of Hep‐2 cells, and decreased survival in the blood, relative to wild type.^110^ This could be attributed to additional phenotypic differences between the strains caused by deletion of global regulators. Notably, the mutant with increased capsule thickness showed even lower adherence to Hep‐2 and poorer survival in whole blood relative to the thin‐capsule mutant.^110^ Similarly, *S. aureus* strains with different capsule thicknesses had no significant difference in virulence in a catheter‐induced rat model of endocarditis.^111^ Capsule thickness also does not have a role in every aspect of an infection and may be a context‐dependent fitness factor. For instance, capsule thickness does not impact intracellular survival of *S. iniae* in macrophages.^112^ In this section, we explore the mechanistic factors involved in CPS chain length determination and its regulation, reported from across major groups of bacteria. An overview of the systems governing CPS chain length is presented in Figure 3.

### Autophosphorylating PCP‐2a/b‐family proteins determine the chain length of Wzy‐dependent CPSs

Polysaccharide copolymerases (PCPs) are a superfamily of integral inner membrane proteins that facilitate the polymerization and export of polysaccharides synthesized by bacterial EPS biosynthetic systems. With few exceptions, PCP proteins generally share common structural features including a TMD formed by N‐terminal (TM1) and C‐terminal (TM2) transmembrane regions and an intermediate periplasmic domain (PD) composed of an extended α‐helical loop region with coiled‐coil motifs.^113^, ^114^, ^115^ However, some PCPs, such as *E. coli* KpsE, only possess TM1 and lack TM2, where the N‐terminus is located in the cytoplasm and the C‐terminal region is located in the periplasm.^116^

PCPs are classified into three major families PCP‐1, PCP‐2, and PCP‐3, based on structural differences and the type of polysaccharide synthesized.^113^ Functionally, PCP‐1 proteins are associated with the biosynthesis and chain length modulation of LPS O‐antigen and enterobacterial common antigen (ECA).^113^ PCP‐2 proteins are involved in the biosynthesis and chain length regulation of high‐molecular weight CPS, while PCP‐3 are primarily associated with ABC transporter‐dependent CPS production.^113^ Given their established role in CPS chain length regulation, the following discussion focuses on the structure and function of PCP‐2 proteins.

The PCP‐2 family which is further divided into PCP‐2a and PCP‐2b subfamilies is unique among PCPs in containing a cytoplasmic tyrosine‐kinase domain at the C‐terminus (Figure 1D). This kinase domain consists of phosphate‐binding Walker‐A (GX_5_GKS/T; X is any residue) and Walker‐B (Φ_4_DX_2_P; Φ is a hydrophobic residue) motifs, along with a putative Walker‐A′ (Φ_4_DXDXR) motif, eventually terminating in a tyrosine‐rich tail.^117^ These Walker motifs collectively provide a platform for ATP‐Mg^2+^ binding and contribute to the ATPase and autokinase functions of the kinase domain. In PCP‐2a proteins, the TMD and kinase domain are encoded as a single protein, while in PCP‐2b, the kinase domain is encoded as a separate protein but requires a complex formation with the TMD to function. Typically, PCP‐2b proteins are part of Gram‐positive EPS biosynthetic systems (e.g., *S. aureus* CapAB and *S. pneumoniae* CpsBC), whereas PCP‐2a are found in Gram‐negative EPS biosynthetic systems (e.g., *A. baumannii* Ptk, *Burkholderia cepacia* BceF, and *E. coli* Wzc).

The kinase domain of PCP2 proteins can form an octameric ring based on the overall phosphorylation status of the C‐terminal tyrosine cluster (Y‐cluster).^118^, ^119^ The number and arrangement of tyrosine residues in the kinase domain are not well‐conserved across bacterial species.^117^ The tyrosine‐rich tail oscillates between states of high (high Y_[P]_) and low (low Y_[P]_) tyrosine phosphorylation (Figure 3C). Phosphorylation is driven by the intrinsic kinase activity of the domain but requires a low‐molecular weight protein tyrosine phosphatase (LMW‐PTP; e.g., *E. coli* and *K. pneumoniae* Wzb) or polymerase and histidinol phosphatase (PHP; e.g., *S. agalactiae* CpsB) to dephosphorylate the residues.^120^

In the low Y_[P]_ state, Wzc enters an octameric closed state (CS) (Figure 3C). CS stability is achieved by intermonomer interaction between the oligomerization interface (I_1_) located in α2 of one monomer and the complementary interface (I_2_) situated in α7 and α9 helices of the adjacent monomer.^117^ Each monomer serves dual functions as (i) an enzyme to trans‐phosphorylate the Y‐cluster of adjacent monomer and (ii) a Y‐cluster substrate for trans‐phosphorylation by another neighboring monomer. At this stage, ATP‐Mg^2+^ are increasingly recruited to the active sites formed by Walker motifs, resulting in several rounds of Y‐cluster phosphorylation. Once the critical threshold of tyrosine phosphorylation is crossed to achieve a state of high Y_[P]_, the increased electrostatic interactions destabilize the octamer and dissociate the kinase domains into their monomeric open state (OS). Furthermore, I1 of each monomer becomes available for engaging an LMW‐PTP or PHP to dephosphorylate the previously phosphorylated tyrosine residues, generating a low Y_[P]_ state primed for another round of association/dissociation. It should be noted that the transition between these two temporal states is not associated with phosphorylation of any specific tyrosine residues, but rather that the overall phosphorylation level of the Y‐cluster must exceed a critical threshold.^117^, ^118^, ^121^

The phospho‐cycling of the PCP2 kinase domain and resultant cyclic oligomerization is proposed to determine CPS chain length in Wzy‐dependent systems (Figure 3C). The consensus among studies involving strategic mutagenesis of the Y‐cluster and Walker motifs is that abolishment or reduced phosphorylation of the Y‐cluster leads to the production of CPS or EPS with increased or more uniform chain length.^99^, ^122^, ^123^, ^124^ In contrast, mutations increasing the phosphorylation of the Y‐cluster leads to polysaccharide products with reduced chain length or variable chain length distribution. It is proposed that the mutations lead to altered product size due to either (i) increased stability of the CS or OS such that the PCP2 remains in the CS or OS for a longer time period, or (ii) a changed critical threshold of phosphorylation required to transition between the CS or OS such that increased or decreased phosphorylation is required to switch the oligomerization state.^117^ PCP2 mutations reported to alter CPS chain length have been observed to naturally occur in clinical isolates and bacteria placed under different environmental stressors (see “Environmental cues modulate the bacterial capsular polysaccharide chain length” section). The molecular mechanism by which the transition of the PCP2 kinase to a monomeric state signals Wzy to terminate the polymerization of the growing CPS chain, thereby defining the glycopolymer chain length remains unclear. In a comparable LPS biosynthetic system, interaction of a PCP‐3 TMD with the WzyB polymerase is shown to be necessary for O‐antigen chain length regulation.^125^ Therefore, we speculate that kinase octamer dissociation induces conformational changes in the PCP‐2 TMD, affecting its interaction with Wzy. This could disengage Wzy from the growing CPS chain and promote translocation of the CPS chain via Wza. However, whether PCP2 interacts with Wzy remains to be determined.

### Intracellular pool of nucleotide‐sugar precursors modulates CPS chain length in synthase‐dependent systems

A PCP2‐like phospho‐cycling system regulating CPS chain length is absent in the synthase‐dependent CPS biosynthesis system. Thus, an alternative CPS chain length controlling mechanism is employed. Specifically, the concentration of nucleotide sugar substrates modulates the chain length of synthase‐dependent CPSs (e.g., *S. pneumoniae* type 3 CPS).^56^, ^126^, ^127^, ^128^ The *S. pneumoniae* type 3 cellubiuronic CPS is composed of alternating β‐1,3‐glucose (Glc) and β‐1,4‐glucuronic acid.^129^ Synthase‐dependent polymerization of *S. pneumoniae* type 3 CPS occurs in two kinetic phases: (i) the transitory phase, in which, a short oligosaccharide–lipid primer is synthesized and weakly associated with the synthase complex, and (ii) the processive phase, in which, the primer is extended one sugar at a time to form long CPS polymers while the polysaccharide is held strongly by the synthase.^54^, ^55^, ^56^ The transition from the oligosaccharide–lipid synthesis phase to the processive phase requires an approximately eight‐sugar oligosaccharide that interacts with the carbohydrate recognition site of the synthase.^55^ Once in the processive phase, the polymer remains strongly associated with the carbohydrate recognition site of the synthase and a high‐molecular weight long chain is synthesized. However, the concentration of nucleotide sugar precursors UDP‐GlcA and UDP‐Glc determines how long the growing polymer and the synthase remain associated and, as a result, determines the chain length of the final CPS polymer.^55^, ^126^, ^127^ In the *S. pneumoniae* cellubiuronan synthase system, higher concentrations of UDP‐GlcA favors a shift from the transitory phase to the highly processive phase leading to the formation of long CPS polymer.^54^, ^55^, ^56^, ^126^, ^127^ Meanwhile, lower concentrations of UDP‐GlcA eject polysaccharide from the carbohydrate recognition site, terminating chain synthesis more frequently.^54^, ^55^, ^56^, ^126^, ^127^ An increase in UDP‐GlcA availability from 1 to 11.5 µM resulted in a more than 20‐fold increase in polymer chain length in an in vitro model of *S. pneumoniae* cellubiuronan CPS synthesis.^56^

Activated sugar substrate concentrations also modulate CPS chain length in *S. pyogenes* and *S. zooepidemicus* hyaluronan synthase systems that polymerize alternating repeats of β‐1,4‐GlcA and β‐1,3‐GlcNAc.^128^, ^130^ In *S. zooepidemicus*, increasing intracellular concentrations of UDP‐GlcNAC by overexpressing the UDP‐GlcNAc biosynthetic enzymes (*hasE* and *hasD*) increased the molecular weight of the hyaluronan CPS. This is an example of substrate‐dependent chain length determination.^128^, ^131^ A similar substrate‐level regulatory mechanism modulates Gram‐positive lipoteichoic acid (LTA) chain length. During LTA synthesis, high concentrations of lipid starter units (diglucosyl diacylglycerol) cause premature dissociation of the growing polymer from the LTA synthase (LtaS) and decreases LTA chain length.^132^

### Environmental cues modulate the bacterial capsular polysaccharide chain length

The fine‐tuning of CPS properties is a key factor in the adaptation of capsulated bacteria to diverse environments.^133^ In many bacterial species, various environmental cues, such as oxygen and carbon dioxide levels, different carbon sources, iron availability, and solute concentration, regulate CPS abundance.^134^, ^135^, ^136^, ^137^, ^138^, ^139^, ^140^, ^141^, ^142^ However, adaptive changes to CPS chain length have been reported in only a few bacterial species, including *A. baumannii*, *K. pneumoniae*, and *S. pneumoniae*.^27^, ^103^, ^126^, ^143^, ^144^ The underlying mechanisms for these changes, which remain largely unclear, are crucial for elucidating bacterial pathogenesis within specific host environments.

Some Gram‐negative bacterial species modulate CPS chain length upon exposure to varying carbon sources, human urine, or host epithelial cells.^27^, ^103^, ^144^ For instance, *K. pneumoniae*, a common urinary tract pathogen, primarily produces long CPS chains with uniform length in nutrient‐rich growth media but shifts to shorter and diverse chain length when exposed to human urine.^27^ Remarkably, urine‐evolved *K. pneumoniae* Wzc variants retain the ability to produce longer CPS chains with uniform length in both growth conditions.^27^ Similarly, in vitro exposure of *A. baumannii* to lung epithelial cells selects for bacterial variants with increased and uniform CPS chain length mediated by spontaneous mutations in *wzc* (also referred to as *ptk*).^103^ These bacterial species with longer and more uniform CPS chain length present as hypermucoviscous colonies, a phenotype associated with increased invasive potential and infection severity.^100^, ^145^, ^146^, ^147^ Environmental factors such as carbon sources, temperature and pH further influence the bacterial mucoviscosity.^27^, ^148^, ^149^ Specialized regulatory proteins, such as the regulator of mucoid phenotype D (RmpD) in *K. pneumoniae* provide an additional layer of control over CPS chain length modulation.^100^, ^150^ In strains encoding RmpD, CPS chain length and mucoviscosity are modulated in an RmpD‐dependent manner by environmental signals including the availability of arginine and sugars.^148^, ^151^ Although the regulatory network is likely more complex, signal‐specific regulators such as the carbon catabolite regulator cyclic AMP receptor protein (CRP) and associated small regulatory RNAs have been implicated in integrating environmental signals to regulate *K. pneumoniae* mucoviscosity and consequently, CPS chain length.^152^ Altogether, these environment‐dependent chain length modulations in Gram‐negative bacteria contribute to enhanced bacterial virulence and persistence by altering the degree of attachment to host epithelial cells and invasion of phagocytic cells.^22^, ^27^, ^103^

In Gram‐positive bacteria, capsule thickness and CPS chain length are modulated by various intrinsic and extrinsic factors. Notably, environmental cues such as carbon sources strongly influence capsule thickness across various streptococcal species, including *S. pneumoniae*, *S. suis*, and *S. iniae*.^26^, ^30^, ^134^, ^143^, ^144^, ^153^, ^154^, ^155^, ^156^, ^157^ In *S. pneumoniae*, CPS chain length is determined by cellular UDP‐sugar precursor levels, with low UDP‐sugar levels correlating with decreased CPS chain length (see “Intracellular pool of nucleotide‐sugar precursors modulates CPS chain length in synthase‐dependent systems” section).^127^ Alterations in carbon sources affect intracellular CPS precursor levels and significantly impact capsule thickness. When cultured in fructose compared to glucose, *S. pneumoniae* shows reduced intracellular concentrations of UDP‐glucose and UDP‐galactose, resulting in decreased capsule thickness.^143^ Decreased UDP‐sugar precursors and reduced capsule thickness are also observed when *S. pneumoniae* is cultured with mannose or GlcNAc compared to glucose or galactose, further supporting a link between CPS precursor availability and capsule thickness or CPS chain length.^144^ Conversely, *S. suis*, a significant zoonotic pathogen in swine, increases capsule thickness in glucose‐rich conditions.^26^ Different carbon sources also alter global transcriptional regulators that affect capsule thickness. High glucose availability activates carbon catabolite protein A (CcpA) which activates transcription of several gene targets including the CPS biosynthetic locus.^26^ CodY, a master regulator in Gram‐positive bacteria, is positively influenced by the flux of branched‐chain amino acids (BCAA). Notably, a CodY deletion decreases capsule thickness in *S. suis* during both exponential and stationary phases.^155^ Conversely, mutations in the virulence‐associated serine/threonine phosphatase 1 (Stp1) lead to increased capsule thickness.^156^, ^158^, ^159^, ^160^, ^161^ Although it remains unclear, Stp1 likely dephosphorylates CPS biosynthesis‐associated targets to influence capsule thickness. Beyond carbon sources, nutritional metal ion pools play a pivotal role in modulating capsule thickness. In *S. pneumoniae*, high Mn/low Zn ratio increases capsule thickness by hyperactivating phosphoglucomutase (Pgm) and likely activating CpsB (PHP) required for dephosphorylation of CpsD tyrosine kinase.^154^ Pgm is an enzyme responsible for generating CPS biosynthesis precursors. The effect of Pgm on capsule thickness is also evident in the zoonotic bacterium *S. iniae*, where deletion of Pgm decreases capsule thickness.^162^ Similar to Gram‐negative bacteria, regulation of capsule thickness or CPS chain length in Gram‐positive bacteria has major implications for bacterial pathogenicity. In *S. pneumoniae* and *S. suis*, environment‐dependent reduction in capsule thickness is associated with increased bacterial adherence to and invasion of host cells, as well as reduced pathogenesis in murine infection models.^26^, ^144^, ^158^

## CONCLUSION

Decades of works from various groups have significantly advanced our understanding of bacterial CPS biosynthesis and its regulatory mechanisms. However, much of these works have centered on the overproduction or underproduction of CPSs, often overlooking the intricate ways bacteria can fine‐tune various CPS properties to improve their fitness within a host. In this review, we summarized current knowledge of the mechanisms governing more nuanced bacterial CPS properties, namely, attachment and chain length control, and highlighted outstanding questions in the field.

The current literature on bacterial CPS chain length and attachment has several limitations. One significant issue encountered is the lack of uniformity in experimental techniques used to study CPS properties. Even within the same bacterial species, some researchers have used microscopy to quantify CPS thickness and abundance while others have used biochemical assays, leading to inconsistencies in data interpretation and complicating the formulation of broad conclusions. Additionally, there is inconsistency in how CPS features are described across studies. For instance, “capsule thickness” is often used interchangeably with “CPS abundance.” Furthermore, capsule thickness is an ambiguous feature and does not clearly explain the individual contributions of CPS chain length, abundance and retainment, especially since bacteria can modulate these features distinctly. These challenges underscore the importance of developing robust, standardized techniques to discretely quantify critical CPS features.

Specific systems governing CPS chain length and attachment are remarkably diverse with significant gaps in our understanding. It is intriguing that CPS‐binding lectin Wzi neither accounts for complete capsule retention nor is it encoded in all Wzy‐dependent systems. This raises the question whether certain bacterial species synthesize compositionally heterogenous CPS that require multiple surface‐associated factors for attachment. Additionally, CPS chain length regulation is not apparent across all CPS biosynthetic systems. For instance, further investigation is required to determine if CPS chains are constitutively synthesized at dysregulated length (*E. coli* K1 CPS) or if a yet‐to‐be identified factor controls their length in ABC transporter‐dependent systems.

The regulation of CPS properties extends well beyond controlling its abundance. Capsulated bacteria respond to environmental cues within the host to finely tune additional CPS properties such as chain length and attachment, which play critical role in enhancing niche‐specific fitness. Despite the significant contribution of these properties to environmental‐ and host‐specific fitness, several aspects of these CPS features remain poorly understood. Continued investigation of molecular mechanisms behind these features could offer promising novel therapeutic targets, especially in the rising global antimicrobial‐resistance landscape.

## AUTHOR CONTRIBUTIONS

Saroj Khadka drafted the manuscript; Saroj Khadka and Laura A. Mike conceptualized the study; Saroj Khadka, Emily L. Kinney, Brooke E. Ryan, and Laura A. Mike were involved in the writing and revision of the manuscript.

## CONFLICT OF INTEREST STATEMENT

The authors declare no conflicts of interest.

## PEER REVIEW

The peer review history for this article is available at https://publons.com/publon/10.1111/nyas.15364.

## Data Availability

Data sharing not applicable to this article as no datasets were generated or analyzed during the current study.
